# Detection of Lung Cancer Cells in Solutions Using a Terahertz Chemical Microscope

**DOI:** 10.3390/s21227631

**Published:** 2021-11-17

**Authors:** Yuichi Yoshida, Xue Ding, Kohei Iwatsuki, Katsuya Taniizumi, Hirofumi Inoue, Jin Wang, Kenji Sakai, Toshihiko Kiwa

**Affiliations:** 1 Graduate School of Interdisciplinary Science and Engineering in Health Systems, Okayama University, Okayama 700-8530, Japan; pjaa6rfb@s.okayama-u.ac.jp (Y.Y.); pm7g9k5d@s.okayama-u.ac.jp (X.D.); prsd3x9h@s.okayama-u.ac.jp (K.I.); p29v8wjz@s.okayama-u.ac.jp (K.T.); wangjin@okayama-u.ac.jp (J.W.); sakai-k@okayama-u.ac.jp (K.S.); 2 Graduate School of Medicine Dentistry and Pharmaceutical Sciences, Okayama University, Okayama 700-8558, Japan; inoue-h1@cc.okayama-u.ac.jp

**Keywords:** terahertz, cancer genomic medicine, cancer cells

## Abstract

Cancer genome analysis has recently attracted attention for personalized cancer treatment. In this treatment, evaluation of the ratio of cancer cells in a specimen tissue is essential for the precise analysis of the genome. Conventionally, the evaluation takes at least two days and depends on the skill of the pathologist. In our group, a terahertz chemical microscope (TCM) was developed to easily and quickly measure the number of cancer cells in a solution. In this study, an antibody was immobilized on a sensing plate using an avidin-biotin reaction to immobilize it for high density and to improve antibody alignment. In addition, as the detected terahertz signals vary depending on the sensitivity of the sensing plate, the sensitivity was evaluated using pH measurement. The result of the cancer cell detection was corrected using the result of pH measurement. These results indicate that a TCM is expected to be an excellent candidate for liquid biopsies in cancer diagnosis.

## 1. Introduction

Cancer genes have been identified using next-generation sequencing (NGS) technology [1]. Therefore, cancer genomic medicine has recently been recognized as a promising option for cancer treatment to reduce the physical burden of cancer patients during treatment. Cancer genomic medicine provides personalized treatment to each patient by precisely analyzing the genomes. The precision of genome analysis largely depends on the ratio of cancer cells to normal cells in a sample specimen tissue. Therefore, quantitative measurements of cancer cells in the sample specimens are essential for analysis. Conventionally, the ratio of cancer cells to normal cells is evaluated as follows [2]: first, the specimen tissue is fixed to make formalin-fixed paraffin-embedded (FFPE) [2,3,4,5,6] by replacing water with formalin, which takes between 24 and 48 h [7]; subsequently, the tissue is degreased with alcohol, followed by paraffin embedding; the tissue is then sliced and stained. The stained tissue is visually observed using an optical microscope by pathologists. However, this evaluation process takes at least 2 days, and largely depends on the skill of the pathologist.

On the contrary, a terahertz wave refers to an electromagnetic wave with the frequency components between 0.1 and 10 THz [8]. Terahertz sources are generally used as optical sources for imaging and spectroscopy [9,10,11,12,13]. Recent progress in terahertz technology has enabled the use of this technology for medical and/or biosocial measurements [9,12,14,15,16].

Previously, our group developed a terahertz chemical microscope (TCM) and proposed to measure chemical reactions in the solution on the sensing plate [17,18,19,20,21]. The TCM can evaluate the number of cancer cells in a solution without the fixing process [19,20]. When using a TCM, the only pretreatment required is to drop the solution containing cancer cells and stir it. A TCM can take measurements within 25 min, including the pretreatment time, and it is therefore expected to be an easier and faster evaluation method. In addition, as the TCM can evaluate the number of cancer cells quantitatively, it can be evaluated without depending on the pathologists’ skill. TCMs can selectively detect cancer cells in solution using the immune reaction of cells and antibodies on the sensing plate. Therefore, it is important to immobilize antibodies on the sensing plate at a higher density without losing antibody function.

In this study, biotin-labeled cytokeratin was conjugated with avidin immobilized on a sensing plate to detect lung cancer cells [19]. Since one avidin molecule can be conjugated with several biotins, there is a higher immobilization of cytokeratin compared to that when the cytokeratin is on the sensing plate. To compensate for the variability of the sensitivity of the sensing plate, the pH dependence of the amplitude of the terahertz wave from the sensing plate and the obtained data were normalized by the pH dependence.

## 2. Detection Principle and Experimental Setup of TCM

**Figure 1:**
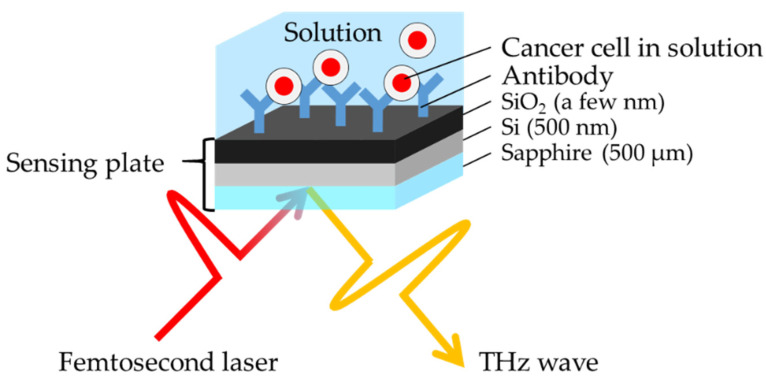
Schematic diagram of the sensing plate and the terahertz wave radiated from the sensing plate.

Figure 1 shows a schematic diagram of the sensing plate used as a terahertz emitter. The sensing plate consisted of a silicon oxide (SiO_2_) film (a few nanometers thick) and a silicon (Si) layer (500 nm thick) on a sapphire substrate (500 µm thick). The dimensions were 10 × 10 mm. A terahertz wave was generated in the Si layer and radiated to the free space by irradiating a femtosecond laser pulse into the sensing plate from the sapphire substrate side. The radiated terahertz wave does not interact with materials on the sensing plate; therefore, the TCM can measure the sample even in the water solution, which absorbs the terahertz wave.

The amplitude of the radiated terahertz wave is expressed as follows:

(1) $$E_{\mathrm{THz}}\left(t\right)\propto \frac{\partial J\left(t\right)}{\partial t}\propto e\frac{\partial n\left(t\right)}{\partial t}v+en\frac{\partial v\left(t\right)}{\partial t},$$

where *E*_THz_(*t*) is the electric field of the terahertz wave, *J*(*t*) is the instantaneous current density in the Si layer, *e* is the elementary charge, *n*(*t*) is the carrier density of the Si layer, and *v*(*t*) is the velocity of the carriers accelerated in the Si layer. The first term in Equation (1) is proportional to the time variation of the carrier density, and the second term is proportional to the time variation of the carrier velocity, i.e., the acceleration of the carriers. Therefore, this equation indicates that *E*_THz_(*t*) is proportional to the electric field in the Si layer, which forms naturally within the depletion layer of the Si layer. Cancer cells in solution react with antibodies immobilized on the sensing plate, and the electric potential is changed by the charge of cancer cells; simultaneously, the electric field in the Si layer changes in magnitude. Thus, the reaction between the cancer cells and antibodies on the sensing plate can be measured by measuring the amplitude of the terahertz wave. Currently, because of the variability in the properties of the sensing plates, calibration of the sensitivity is essential for accurate measurements.

According to [21], the sensing plate can measure pH values in solutions because protons react with the SiO_2_ surface of the sensing plate according to the following equation, which changes the electric field of the sensing plates:

(2) $${\mathrm{SiOH}}_{2}^{+}↔\mathrm{SiOH}+H^{+}$$

(3) $$\mathrm{SiOH}↔{\mathrm{SiO}}^{-}+H^{+}$$

To evaluate the sensitivity of each sensing plate, buffer solutions with pH values of 10.01 and 1.68 were initially measured to calibrate the measured amplitude of the terahertz waves.

**Figure 2:**
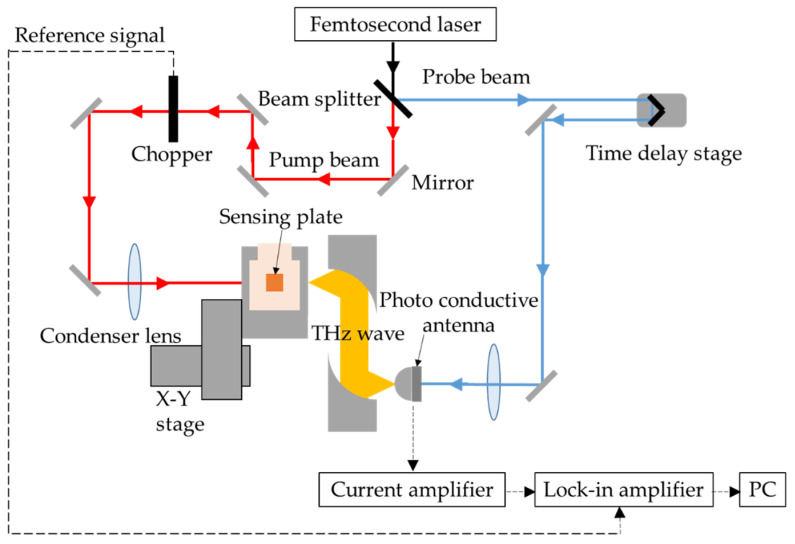
Optical system diagram of the TCM.

Figure 2 shows the optical system diagram of the TCM. A femtosecond laser (FemtoFiber Ultra 780, TOPTICA Photonics AG, Munich, Germany) was used as the optical source. The center of wavelength and pulse width were 780 nm and 100 fs, respectively, at full width at half-maximum (FWHM). The average output power was 500 mW with a repetition frequency of 82 MHz, which corresponds to 6.25 nJ/pulse. The spot size was approximately 1 mm in diameter on the sensing plate. The femtosecond laser was divided into a pump beam and probe beam by a beam splitter. The pump beam was focused on the substrate side of the sensing plate using a condenser lens to generate a terahertz wave. The terahertz wave was collimated and focused on a terahertz detector by a pair of off-axis parabolic mirrors. A bow-tie-type photoconductive antenna (PCA) made from a low-temperature-grown GaAs was used as the terahertz detector. The probe beam was focused on the PCA in order to trigger it, and the arrival time of the probe beam to the PCA was optimized by a time delay stage to detect the peak amplitude of the terahertz waves. The intensity of the pump beam was modulated by an optical chopper at a frequency of 2 kHz; thus, the signal was lock-in detected after being amplified by a current amplifier with a magnification factor of 10^8^ V/A. The sensing plate was mounted on the stepping-motor-drive *x-y* stage to scan the femtosecond laser across the surface of the sensing plate, to visualize the amplitude distribution of the radiated terahertz wave.

## 3. Materials and Methods

**Figure 3:**
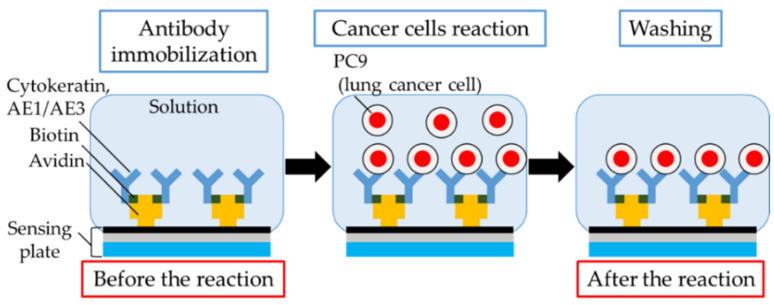
Schematic diagram of an experimental process to detect cancer cells in solution.

Figure 3 shows the process of measuring the cancer cells in solution. First, avidins (affinity-purified, Vector Laboratories, Inc., Burlingame, CA, USA) were immobilized on the SiO_2_ film of the sensing plate using the amine coupling method [22,23]; the surface of the SiO_2_ film on the sensing plate was ultrasonically cleaned for 2 min with acetone (99.5%, Sigma-Aldrich Japan G.K, Tokyo, Japan) and ethanol (99.5%, Hayashi Pure Chemical Ind., Ltd., Osaka, Japan). The SiO_2_ film was modified with ester groups for 1 h at −21 °C, soaking in toluene containing 0.5 mM 2-(carbomethoxy)ethyl-trichlorosilane (FUJIFILM Wako Pure Chemical Corporation, Osaka, Japan). The ester groups were carboxylate soaked in hydrochloric acid (35%–37%, FUJIFILM Wako Pure Chemical Corporation, Osaka, Japan). The carboxyl groups were activated by soaking in pH 7.4 solution. This solution consisted of pH 7.4 PBS (Thermo Fisher Scientific Inc., Waltham, MA, USA), containing 3 mM N-hydroxy succinimide (NHS) (98.0%–102.0%, FUJIFILM Wako Pure Chemical Corporation, Osaka, Japan) and 1 mM 1-Ethyl-3-(3-dimethylaminopropyl) carbodiimide hydrochloride (EDC) (>99.9%, FUJIFILM Wako Pure Chemical Corporation, Osaka, Japan) to facilitate the reaction of the amino groups in avidin and the carboxyl groups within the SiO_2_ film on the sensing plate. Avidin (10 µg/mL) was added to each well of 30 µL and was immobilized on the SiO_2_ film on the sensing plate for 24 h at 4 °C. To prevent non-specific adhesion of proteins on the sensing plate, 1 mM 2-amineethanol (>99.0%, Tokyo Chemical Industry Co., Ltd., Tokyo, Japan) was combined with the pH 7.4 PBS and was applied to each well of 30 µL. It was then reacted with the surface of the sensing plate for 15 min at 18–25 °C. Subsequently, biotin-labeled cytokeratin AE1/AE3 (Protein A or G purified, Novus Biologicals, Briarwood Avenue, Centennial, CO, USA) was conjugated as an antibody with the avidins by applying the solution with an AE1/AE3 concentration of 10 µg/mL and shaking the sensing plate using an orbital shaker for 30 min at 18–25 °C, with a rotation speed of 45 rpm.

**Figure 4:**
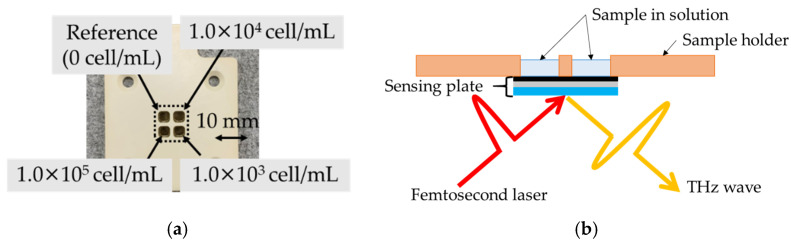
(**a**) Sensing plate with four wells. The concentrations of cells in each well are indicated in the photo. (**b**) Cross-section diagram of the sensing plate with wells.

After immobilizing the AE1/AE3, the distribution of the terahertz amplitude was measured as a background signal. In this study, cultivated human lung adenocarcinoma cells (PC9) were used as sample cells. The concentrations of cancer cells in the culture medium were 10^3^, 10^4^, and 10^5^ cell/mL, which were measured using an automated cell counter (Countess 2, Thermo Fisher Scientific, Tokyo, Japan). Figure 4a,b show the sensing plate with four wells and the cross-section of the sensing plate, respectively. The sample solutions with cells were infected in each well at a volume of 30 µL. The concentration of cells is shown in Figure 4a. The cells were incubated with the immobilized antibodies for 15 min at room temperature at 45 rpm. Thereafter, the non-reacted cancer cells were removed by washing 10 times. The distribution of the terahertz amplitude after reacting the cancer cells was measured using the TCM.

## 4. Results and Discussion

**Figure 5:**
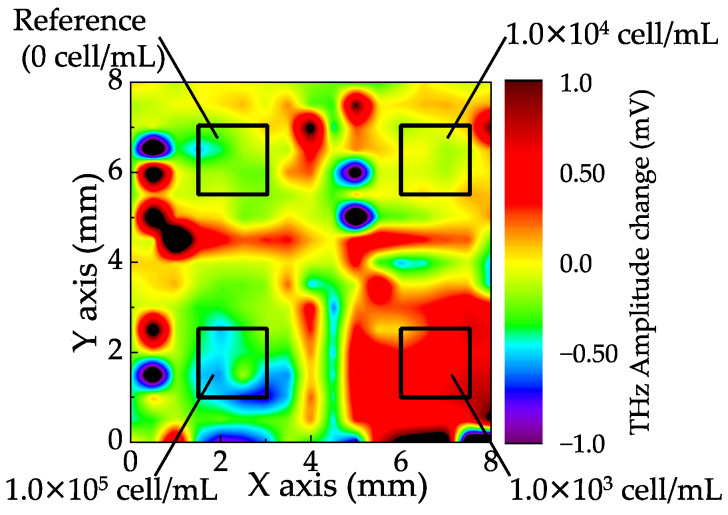
Distribution of the change in terahertz amplitude on the sensing plate as shown by the black rectangles in Figure 4a between before and after the reaction of PC9.

**Figure 6:**
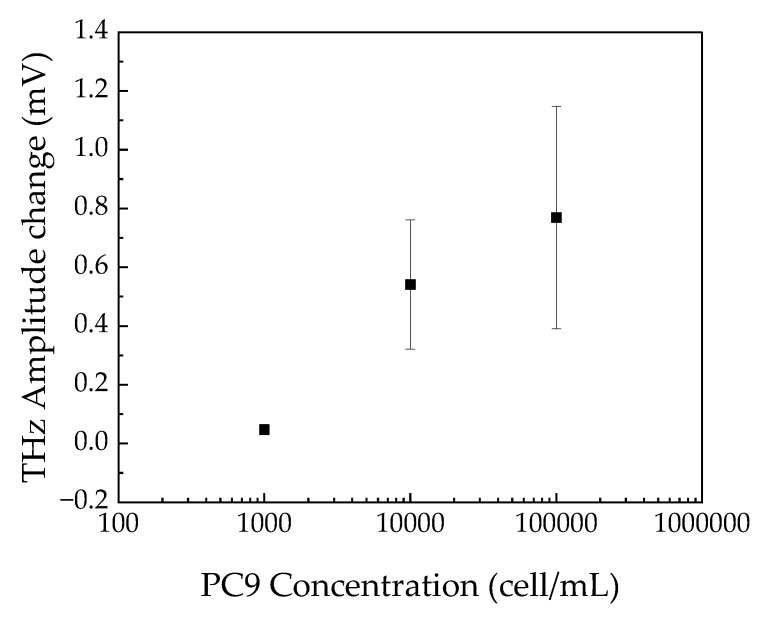
Average value of change in terahertz amplitude for each well in the black rectangles shown in Figure 5 as a function of the concentration of PC9 in solution. The error bar is the standard deviation for the data obtained by three independent sensing plates. The vertical axis was offset by fitting the data.

Figure 5 shows the distribution of the change in the terahertz amplitude on the sensing plate after the reactions, which was obtained by subtracting the data from the background. Black rectangles in the image represent the area of the wells. The total measurement time of the image was approximately 20 min, which was determined by the number of points to measure and the signal-to-noise ratio. The terahertz amplitude was increased by increasing the concentration of cells in the wells. There is a clear irregularity of the terahertz distributions in each well, which is because of the non-uniformity of the reactions. Therefore, the averaged values of the terahertz amplitude in each well were calculated and plotted as a function of the concentration of cells in solution, as shown in Figure 6. The error bar represents the standard division of the data measured by the three different sensing plates. To cancel the offset along the vertical axis, the data were fitted by a double exponential function, given by the following equation:

(4) $$S=S_{0}+A_{1}\left(1-e^{-\frac{c}{t_{1}}}\right)+A_{2}\left(1-e^{-\frac{c}{t_{2}}}\right),$$

where *S* is the average change in terahertz amplitude; *S*_0_ is the terahertz amplitude at 0 cell/mL; *C* is the concentration of cancer cells; and *A*_1_, *A*_2_, *t*_1_, and *t*_2_ are constants to be determined. After determining the value of *S*_0_, the data were subtracted from *S*_0_. Thus, we found that the changes in the terahertz amplitude from the background were 0.047 ± 0.018, 0.54 ± 0.22, and 0.77 ± 0.38 mV for 10^3^, 10^4^, and 10^5^ cell/mL, respectively.

**Figure 7:**
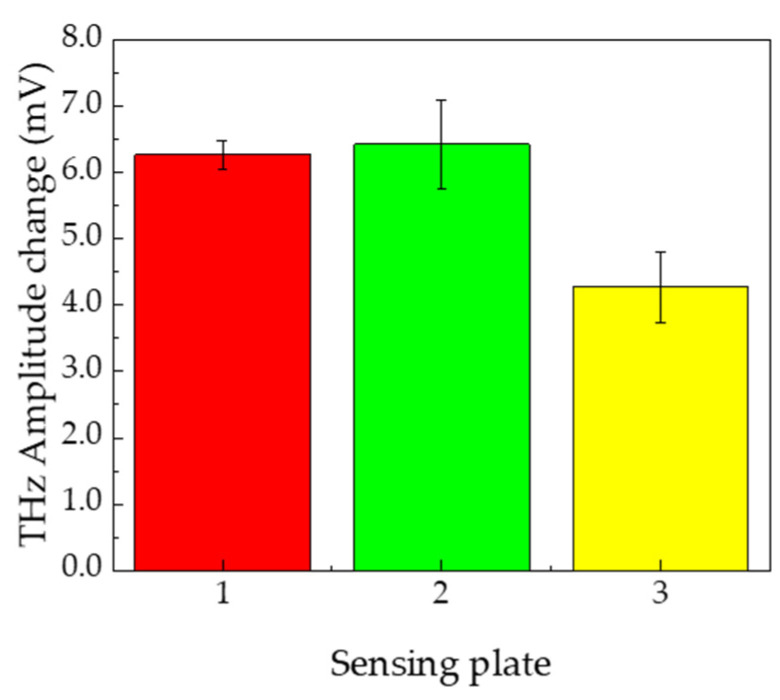
Average value of the change in terahertz amplitude in four wells between pH 1.68 and 10.01, for three independent sensing plates. The error bar shows the standard deviation of the average change in terahertz amplitude of the four wells on the same sensing plate. This indicates that the sensitivity was slightly deviated on the same sensing plate.

Figure 7 shows the change in terahertz amplitude between pH 1.68 and 10.01 for the three sensing plates. The value was calculated by averaging the values of the four wells of each sensing plate.

The error bars in Figure 7 indicate the standard deviation of the values of the four wells in the same sensing plate. The change in terahertz amplitude between pH 1.68 and 10.01 was 6.27 ± 0.21, 6.43 ± 0.66, and 4.27 ± 0.54 mV for sensing plates 1, 2, and 3, respectively. It is clear that the sensitivity of each sensing plate varies, owing to the variation in the properties of the sensing plates, which ultimately leads to a variation in the sensitivity of cancer cell detection. Therefore, the results shown in Figure 6 may be affected by the differences in the sensing plates. The data shown in Figure 6 was consequently normalized by the average value of the change in the terahertz amplitude, the results of which are shown in Figure 7.

**Figure 8:**
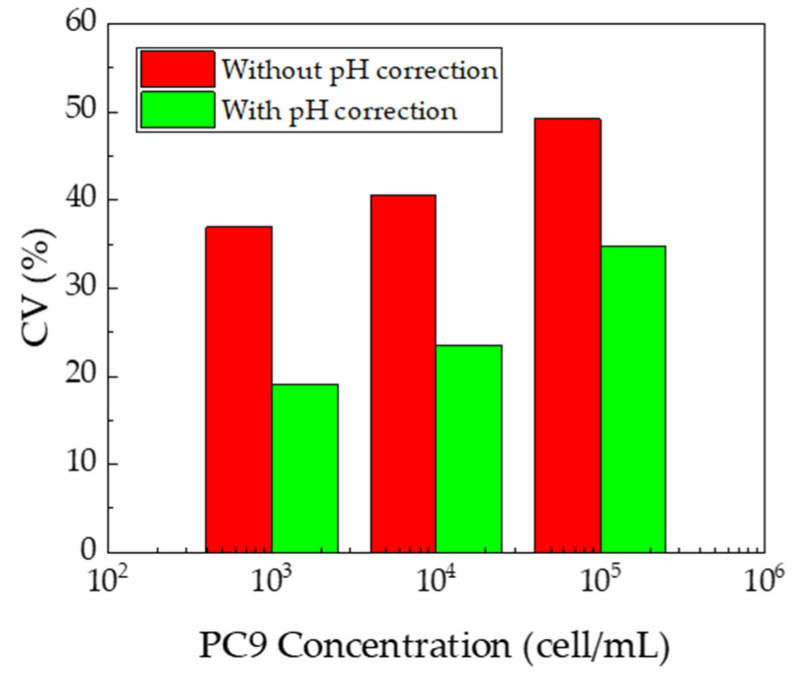
CV in each concentration of PC9.

To study the normalization effect on cancer detection, the coefficient of variation (*CV*) was calculated and is plotted in Figure 8. *CV* was calculated using the following equation:

(5) $$CV=\frac{S_{\mathrm{SD}}}{S_{\mathrm{AVG}}}\times 100\;$$

where *S*_SD_ is the standard deviation of the change in terahertz amplitude and *S*_AVG_ is the average change in the terahertz amplitude. Without pH correction, the *CV* values were 36.9%, 40.6% and 49.2% for 10^3^, 10^4^ and 10^5^ cell/mL, respectively. Contrarily, with pH compensation, the values were 19.1%, 23.4% and 34.7% for 10^3^, 10^4^ and 10^5^ cell/mL, respectively. Therefore, the variation was reduced by a factor of 1.9, 1.7 and 1.4 at 10^3^, 10^4^ and 10^5^ cell/mL, respectively. This result indicates that pH compensation of the sensitivity of the sensing plates was effective for accurate measurement of cancer cells.

## 5. Conclusions

Lung cancer cells in a solution were measured with an antibody immobilized on the sensing plate using the avidin-biotin reaction. The sensitivity of the sensing plate was evaluated using the change in the terahertz amplitude in the pH measurement. The result of lung cancer cell detection was corrected using the results of the pH measurements. The sensitivity correction of the sensing plate was found to be effective for detecting cancer cells. These results indicate that a TCM is expected to be an excellent candidate for liquid biopsies in cancer diagnoses.

## Data Availability

The data that support the findings of this study are available from the corresponding author, [T.K.], upon reasonable request.

## References

[1] The International Cancer Genome Consortium (2010). International Network of Cancer Genome Projects. Nature.

[2] Gaffney E.F., Riegman P.H., Grizzle W.E., Watson P.H. (2018). Factors That Drive the Increasing Use of FFPE Tissue in Basic and Translational Cancer Research. Biotech. Histochem..

[3] Corless C.L., Spellman P.T. (2012). Tackling Formalin-Fixed, Paraffin-Embedded Tumor Tissue with Next-Generation Sequencing. Cancer Discov..

[4] Kou T., Kanai M., Matsumoto S., Okuno Y., Muto M. (2016). The Possibility of Clinical Sequencing in the Management of Cancer. Jpn. J. Clin. Oncol..

[5] Bolognesi C., Forcato C., Buson G., Fontana F., Mangano C., Doffini A., Sero V., Lanzellotto R., Signorini G., Calanca A. (2016). Digital Sorting of Pure Cell Populations Enables Unambiguous Genetic Analysis of Heterogeneous Formalin-Fixed Paraffin-Embedded Tumors by Next Generation Sequencing. Sci. Rep..

[6] Kerick M., Isau M., Timmermann B., Sültmann H., Herwig R., Krobitsch S., Schaefer G., Verdorfer I., Bartsch G., Klocker H. (2011). Targeted High Throughput Sequencing in Clinical Cancer Settings: Formaldehyde Fixed-Paraffin Embedded (FFPE) Tumor Tissues, Input Amount and Tumor Heterogeneity. BMC Med. Genom..

[7] Thavarajah R., Mudimbaimannar V.K., Rao U.K., Ranganathan K., Elizabeth J. (2012). Chemical and Physical Basics of Routine Formaldehyde Fixation. J. Oral. Maxillofac. Pathol..

[8] Tonouchi M. (2007). Cutting-edge terahertz technology. Nat. Photon..

[9] Kawase K., Ogawa Y., Watanabe Y., Inoue H. (2003). Non-destructive terahertz imaging of illicit drugs using spectral fingerprints. Opt. Express.

[10] Fukunaga K., Hosako I., Kohdzuma Y., Koezuka T., Kim M.J., Ikari T., Du X. (2010). Terahertz analysis of an East Asian historical mural painting. J. Eur. Opt. Soc.-Rapid.

[11] Dorney T.D., Baraniuk R.G., Mittleman D.M. (2001). Material parameter estimation with terahertz time-domain spectroscopy. J. Opt. Soc. Am. A.

[12] Hu B.B., Nuss M.C. (1995). Imaging with Terahertz Waves. Opt. Lett..

[13] Nagashima T., Hangyo M. (2005). Evaluation of complex optical constants of semiconductor wafers using terahertz ellipsometry. Springer Ser. Chem. Phys..

[14] Weissleder R. (2006). Molecular imaging in cancer. Science.

[15] Markelz A.G. (2008). Terahertz dielectric sensitivity to biomolecular structure and function. IEEE J. Sel. Top. Quant..

[16] Cheon H., Yang H.J., Lee S.H., Kim Y.A., Son J.H. (2016). Terahertz molecular resonance of cancer DNA. Sci. Rep..

[17] Kiwa T., Oka S., Kondo J., Kawayama I., Yamada H., Tonouchi M., Tsukada K. (2007). A Terahertz Chemical Microscope to Visualize Chemical Concentrations in Microfluidic Chips. Jpn. J. Appl. Phys..

[18] Kiwa T., Kondo J., Oka S., Kawayama I., Yamada H., Tonouchi M., Tsukada K. (2008). Chemical Sensing Plate with a Laser-Terahertz Monitoring System. Appl. Opt..

[19] Hassan E.M., Mohamed A., DeRosa M.C., Willmore W.G., Hanaoka Y., Kiwa T., Ozaki T. (2019). High-Sensitivity Detection of Metastatic Breast Cancer Cells via Terahertz Chemical Microscopy Using Aptamers. Sens. Actuators B Chem..

[20] Nahar S., Mohamed A., Ropagnol X., Hassanpour A., Kiwa T., Ozaki T., Gauthier M.A. (2021). Noninvasive, label-free, and quantitative monitoring of lipase kinetics using terahertz emission technology. Biotechnol. Bioeng..

[21] Kiwa T., Kamiya T., Morimoto T., Fujiwara K., Maeno Y., Akiwa Y., Iida M., Kuroda T., Sakai K., Nose H. (2019). Imaging of Chemical Reactions Using a Terahertz Chemical Microscope. Photonics.

[22] Tero R., Misawa N., Watanabe H., Yamamura S., Nambu S., Nonogaki Y., Urisu T. (2005). Fabrication of Avidin Single Molecular Layer on Silicon Oxide Surfaces and Formation of Tethered Lipid Bilayer Membranes. J. Surf. Sci. Nanotechnol..

[23] Misawa N., Yamamura S., Yong-Hoon K., Tero R., Nonogaki Y., Urisu T. (2006). Orientation of Avidin Molecules Immobilized on COOH-Modified SiO_2_/Si(1 0 0). Surfaces.

